# Study on the Effect of Ginsenosides Rb on Blood of Tumor Mice

**DOI:** 10.1155/2019/5476076

**Published:** 2019-08-18

**Authors:** Mengmeng Zheng, Wenxiu Zheng, Wei Wang, Hong Guo, Hui Cao, Xiaowei Cui, Shanshan Wang, Chunchao Han

**Affiliations:** School of Pharmacy, Shandong University of Traditional Chinese Medicine, Jinan 250355, China

## Abstract

**Objective:**

The blood of cancer patients is in a state of hypercoagulability, easily leading to thrombosis. Anemia is also a complication of tumors. Anemia and thrombosis affect the treatment of tumor patients.

**Methods:**

Ginsenosides Rb were extracted from the stems and leaves of American ginseng using water-saturated ethanol and ethyl acetate in silica gel column. Tumor mice model was established by injecting H_22_ hepatocellular carcinoma cells into the axilla of mice. Mice were randomly divided into 6 groups: normal control group, model control group, positive control group, low dose group (7 mg/kg), middle dose group (14 mg/kg), and high dose group (35 mg/kg). After 18 days, the blood was obtained by picking the eyeball of mice. The levels of red blood cells (RBC), hemoglobin (HGB), neutrophils/lymphocytes radio (NLR), platelets (PLT), platelet distribution width (PDW), fibrinogen (FIB), and D-Dimer (D-D) were measured and compared in each group of mice.

**Results:**

The content of obtained ginsenosides Rb reached 90.05%. This extraction process was simple and reliable. Middle dose of ginsenosides Rb could significantly increase RBC and HGB levels (P<0.05). Moreover, ginsenosides Rb could significantly reduce NLR, PLT, PDW, FIB, and D-D (P<0.01).

**Conclusion:**

ginsenosides Rb could significantly improve anaemia and hypercoagulation of blood in cancer mice. Ginsenosides Rb are a potential anticoagulant and antianemia drug in treating cancer.

## 1. Introduction

**Figure 1:**
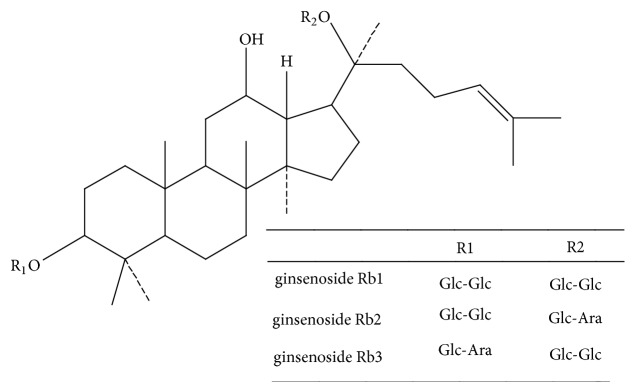
Chemical structural formula of ginsenosides Rb1, Rb2, and Rb3.

American ginseng (*Panax quinquefolium *L.) is a perennial herb of the genus panax quinquefoliaceae, originating from the United States and Canada [1, 2]. American ginseng has been used for several thousand years with functions of adjusting immunity, antitumor, regulating blood sugar level and protecting nervous system [3–7]. Ginsenosides are the major active ingredients of American ginseng, and are extracted from roots, fruits, stems and leaves of American ginseng. According to the difference in the position and quantity of sugar moiety, ginsenosides are divided into three types: A-Panaxadiol group (e.g., Rb1, Rb2, Rb3, Rc, Rd, Rg3, and Rh2), B-Panaxtriol group (e.g., Re, Rg1, Rg2, and Rh1), and C-Oleanolic acid group (e.g., Ro) [8, 9]. Ginsenosides Rb1, Rb2, and Rb3 belong to panaxadiol saponin (Figure 1). Ginsenosides Rb1, Rb2 and Rb3 have many biological activity, such as, anti-myocardial ischemia-reperfusion injury, neuroprotection, and anti-aging [10–12]. Studies of ginsenosides Rb mainly focus on myocardial protection [13]. Few studies have been done on tumor and blood of ginsenosides Rb.

With the development of society, the incidence and mortality of tumors have increased year by year. Studies have shown that tumors can break the balance of coagulation, anticoagulation, and fibrinolysis systems in the body through multiple mechanisms, thus making the blood in a hypercoagulable state [14, 15]. The hypercoagulable state of blood will in turn stimulate the growth and metastasis of tumors and promote their development. Besides, hypercoagulable state of blood can lead to thrombosis, and the incidence of thromboembolic events in tumor patients is 7 times that of normal people [16]. The incidence of venous thromboembolism (VTE) in cancer patients is as high as 20%, and it ranks second in the cause of death among cancer patients [17, 18]. Therefore, anticoagulation therapy has an important significance to reduce the metastasis of malignant tumors and prolong the survival time of patients [19]. NLR, PLT and PDW can be used to evaluate the hypercoagulability of blood [20, 21]. In addition, tumor-associated anemia is one of the common complications in the treatment of cancer patients [22]. Although blood transfusion can partially alleviate tumor-related anemia, there is also the possibility of infection and tumor recurrence [23]. Therefore, improving anaemia and hypercoagulation is beneficial to the treatment of cancer patients.

In the experiment, ginsenosides Rb were extracted from the stems and leaves of American ginseng using water-saturated ethanol and ethyl acetate as eluents. The content of ginsenosides Rb was measured by HPLC. The tumor mice model was established by injecting H_22_ hepatocellular carcinoma cells into the axilla of mice. After 18 days, the blood was obtained by picking the eyeball of mice. The levels of RBC, HGB, NLR, PLT, PDW, FIB, and D-D were measured to study on the effect of ginsenosides Rb on hypercoagulation of blood and Anemia in liver cancer mice.

## 2. Materials and Methods

### 2.1. Chemicals and Reagents

Ginsenosides Rb1, Rb2, Rb3 (purity>98%) were purchased from Aladdin industrial Co., Ltd (Shanghai, China). Ethyl acetate was obtained from Fuyu Fine Chemical Co., Ltd (Tianjin, China). Acetonitrile was obtained from Cinc High Purity Solvents Co., Ltd. (Shanghai, China). Methyl alcohol was purchased from Sinopharm chemical reagent Co., Ltd. (Shanghai, China).

### 2.2. Animal

Female Kunming mice (18-22 g) were purchased from Pengyue laboratory animal Co., Ltd (Jinan, China), License no.: SCXK (lu)20180004. Breeding environment: room temperature was 23°C to 25°C, 35-65% RH. Before the experiment, the animals were acclimatized for a week.

### 2.3. Extraction of Ginsenosides Rb

The total saponins of stem and leaves of American ginseng have been prepared in the early stage of our laboratory [24]. The content of total saponins was 76.51%. 200-300 purpose silica gel was used to separate and purify ginsenosides Rb. The saponin was placed on the upper layer and the 200-300 purpose silica gel was on the lower layer of the silica gel column. The ratio of eluent was ethyl acetate: ethanol: water was 6:1:2 (v/v/v). According to the TLC, the eluent was collected with the same Rf of standard ginsenoside Rb3. The eluent was concentrated and dry.

### 2.4. HPLC Detect the Content of Ginsenosides Rb

#### 2.4.1. The Standard Curve of Ginsenosides Rb1, Rb2 and Rb3

The ginsenoside Rb3 standard was prepared with the following concentrations: 100, 150, 200, 250, and 300 *μ*g/mL. The ginsenosides Rb2 and Rb1 standards were prepared with the following concentrations: 20, 30, 40, 50, and 60 *μ*g/mL, respectively. The absorbancy of ginsenosides Rb1, Rb2, and Rb3 standards was detected under 203 nm by HPLC.

#### 2.4.2. Preparation of Sample Solution

The test product was made to 250 *μ*g/mL with methanol, filtered with 0.45 *μ*m filter membrane.

#### 2.4.3. Chromatographic Condition

Shimazu LC-16. Detector: UV. Chromatographic column: Welch Ultimate XB-C18 (5 *μ*m, 4.6×250 mm). Determine wavelength: 203 nm. Mobile phase: water (A) and acetonitrile (B). Flow rate: 1.0 mL·min^−1^. The sample amount: 20 *μ*L. The gradient elution conditions are shown in Table 1.

### 2.5. Animals Experiments

#### 2.5.1. Preparation of H_22_ Transplantation Mice Model

The mice were killed, which were inoculated with H_22_ tumor cell in abdominal cavity for 8 days. After disinfection, ascites were extracted from the abdominal cavity of the mice. The color of ascites was milky white. Ascites were diluted with saline. The number of H_22_ cells was adjusted to 1×10^7^/mL. Then, diluted ascites were injected into the underarms of the mice, 0.2 mL per mice.

#### 2.5.2. Animal Subgroup and Drug Administration

Female mice were divided into six groups (ten mice per group): normal control group, model control group, positive control group, low dose group, middle dose group and high dose group. The mice in treatment group were intragastrically administered with ginsenosides Rb 7 mg/kg/d, 14 mg/kg/d, and 35 mg/kg/d, respectively. The mice in positive control group were injected intraperitoneally with 30 mg/kg cyclophosphamide every day [25]. The mice in model control group and the control group were given the same volume of normal saline each day. After 18 days, blood was collected from the eyeballs. The following items were tested: RBC, HGB, NLR, PLT, PDW, FIB, and D-D.

### 2.6. Statistical Analysis

Data were analysed by a one-way ANOVA using the statistical software SPSS 22.0. All data were presented as means±SEM, and P-values of 0.05 or less were considered to be statistically significant.

## 3. Results and Discussion

### 3.1. The Content of Ginsenosides Rb in Sample

**Figure 2:**
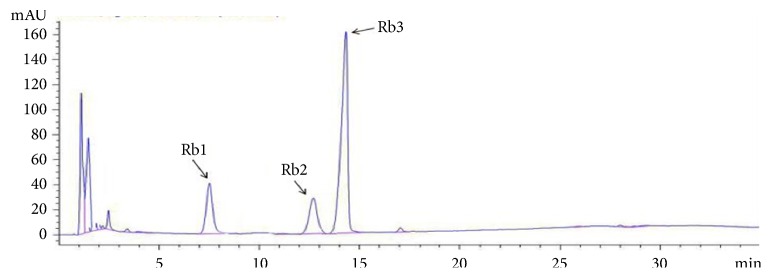
HPLC chromatogram of ginsenosides Rb.

Stems and leaves of American ginseng are the huge resources of the American ginseng. Stems and leaves of American ginseng are often thrown away, leading waste and environmental pollution. In this experiment, the total saponins were extracted from the leaves and stems of American ginseng. In addition, ginsenosides Rb were obtained by water-saturated ethyl acetate and ethanol in silica gel column. This method can not only make rational use of resources, but also has simple process. The HPLC spectrum of ginsenosides Rb was shown in Figure 2. The regression equation of ginsenosides Rb1 was y=5342.1x + 27121, R^2^=0.9994. The regression equation of ginsenosides Rb2 was y=5472.7x + 37704, R^2^=0.9996. The regression equation of ginsenosides Rb3 was y=5715.1 + 46933, R^2^=1.0000. After analysis, the relative content of ginsenosides Rb1, Rb2 and Rb3 was 10.62%, 9.42% and 70.46%, respectively. So, the content of ginsenosides Rb was 90.05%. And the yield of ginsenosides Rb in stems and leaves of American ginseng was 10.7 mg/g. This method is simple and suitable for industrial production. The results showed that the content of ginsenoside Rb3 was significantly greater than that of ginsenosides Rb1 and Rb2. Under this condition, the content of ginsenoside Rb3 was the highest after 5 experiments. When this result occurred, we suspected that the ginsenoside Rb3 was more polar than ginsenosides Rb1 and Rb2. Under this elution condition, ginsenoside Rb3 was eluted first, and the elution condition was favorable for the desorption of ginsenoside Rb3.

### 3.2. Effects of Ginsenosides Rb on Red Blood Cells and Hemoglobin

Chemotherapy is the main treatment for patients with malignant tumors. However, chemotherapy can cause bone marrow suppression in cancer patients, causing anemia [26, 27]. Effective control and treatment of anemia in cancer patients can prolong patient survival. So, the relationship between ginsenosides Rb and anemia was studied. Red blood cells (RBC) and hemoglobin (HGB) are commonly used to evaluate the anaemia. Effects of ginsenosides Rb on RBC and HGB were shown in Table 2. Compared with the normal control group, the contents of RBC and HGB in the model and positive group were significantly decreased (*P<0.05 or P<0.01*). This result was further proved that anemia might occur in patients with cancer and anemia symptoms worsen after chemotherapy. Compared with the model control group, the contents of RBC and HGB in the treatment group were increased. The content of RBC in middle dose group was significantly increased (*P<0.05*). Besides, the content of HGB in middle dose group was also significantly increased (*P<0.05*). This results showed that ginsenosides Rb could improve the symptoms of anemia in cancer patients.

### 3.3. Effects of Ginsenosides Rb on Neutrophils/Lymphocytes Radio, Platelets, and Platelet Distribution Width

Lymphocytes are major members of the immune system and participate in the apoptosis and destruction of tumor cells. The level of lymphocytes is decreased in cancer patents. Decreased level of lymphocytes indicates that the body's immune response is limited. To the opposite, the content of neutrophils is increased in cancer patents. Elevated neutrophils is beneficial for tumor metastasis and invasion. So, the higher the ratio of neutrophils/lymphocytes (NLR), the more immune the body is inhibited [28]. And increased NLR indicates that blood is highly coagulated [20]. What is more, study have found that the numbers of PLT and PDW were increased in cancer patients, suggesting that the blood was highly coagulated [20, 21]. Effects of ginsenosides Rb on NLR, PLT and PDW were shown in Table 3. In this experiment, compared with the normal control group, the levels of NLR, PLT and PDW were significantly increased (*P<0.01 or P<0.05*). However, different doses of ginsenosides Rb could significantly reduce NLR (*P<0.01*). In addition, low dose and middle dose of ginsenosides Rb could reduce PLT. What's more, different doses of ginsenosides Rb could both reduce PDW. Middle dose and high dose could significantly reduce PDW (*P<0.05*). This results further showed that ginsenosides Rb could improve the hypercoagulability state of blood in tumor mice.

### 3.4. Effects of Ginsenosides Rb on Fibrinogen and D-Dimer

As a traditional coagulation parameter, fibrinogen (FIB) and D-Dimer (D-D) are important indexes for evaluating the hypercoagulability state of blood [29, 30]. FIB is a protein with coagulation function and has become an effective indicator for predicting the risk of thrombosis. And D-D can reflect the body's blood hypercoagulability and thrombosis. Effects of ginsenosides Rb on FIB and D-D were shown in Table 4. Compared with normal control group, the levels of FIB and D-D increased significantly in the model group (*P<0.05*). However, low dose and middle dose of ginsenosides Rb could significantly decreased the levels of FIB and D-D in tumor mice. The results suggested that ginsenosides Rb could be a potential anticoagulant in the treatment of tumors.

## 4. Conclusion

In conclusion, ginsenosides Rb were obtained by water-saturated ethyl acetate and ethanol in silica gel column and the relative content of obtained ginsenosides Rb reached 90.05%. This method was simple and suitable for industrial production. Furthermore, ginsenosides Rb could markedly increase the contents of RBC and HGB in tumor mice. Moreover, ginsenosides Rb could significantly increase the levels of NLR, PLT, PDW, FIB, and D-D. Therefore, ginsenosides Rb may become a drug to prevent tumor-related hypercoagulability and anemia.

We have only conducted a preliminary study on the blood of tumor mice. Many researches require us to continue to study in depth. Study found that erythropoietin could help treat tumor-related anemia and improve anemia and sleep quality of patient [31, 32]. The relationship between erythropoietin and ginsenosides Rb will be explored in our future research. In addition, we also studied the changes of blood rheology in tumor rats.

## Figures and Tables

**Table 1:** Gradient elution process.

Time/min	A (water)%	B (acetonitrile)%
0	70	30
10	70	30
25	56	44

**Table 2:** Effects of ginsenosides Rb on RBC and HGB ($\overset{-}{x}$±s, n=10).

Group	Dose	RBC (10^12^/L)	HGB
Normal control	----	9.44±16.84	159.00±16.84
Model control	----	9.20±0.36	142.25±9.22^##^
Positive control	30 mg/kg	8.83±0.39^#^	142.33±9.76^##^
Low dose	7 mg/kg	9.60±0.85	146.75±11.59
Middle dose	14 mg/kg	9.91±0.53^*∗*^	154.00±10.49^*∗*^
High dose	35 mg/kg	9.29±0.62	144.86±9.17

Note: Data are presented as mean±S.E.M. for each group. ^#^*P <0.05 *and^##^*P<0.01:* significant difference compared with normal control group. ^*∗*^*P<0.05 *means significant difference compared with model group.

**Table 3:** Effects of ginsenosides Rb on NLR, PLT, and PDW ($\overset{-}{x}$±s, n=10).

Group	Dose	NLR	PLT (10^9^/L)	PDW
Normal control	----	0.34±0.08	1580.20±159.94	16.10±0.23
Model control	----	0.89±0.05^###^	1907.71±219.87^#^	16.37±1.01^#^
Positive control	30 mg/kg	0.62±0.10^*∗*^	1732.44±193.27	16.37±0.19
Low dose	7 mg/kg	0.46±0.06^*∗∗*^	1826.75±151.27	16.23±0.23
Middle dose	14 mg/kg	0.42±0.06^*∗∗*^	1797.80±134.91	16.15±0.15^*∗*^
High dose	35 mg/kg	0.51±0.04^*∗∗*^	1930.14±136.34	16.09±0.08^*∗*^

Note: Data are presented as mean±S.E.M. for each group. ^#^*P<0.05 *and^###^*P<0.001*: significant difference compared with normal control group. ^*∗*^*P<0.05 *and ^*∗∗*^*P<0.01*:significant difference compared with model control group.

**Table 4:** Effects of ginsenosides Rb on FIB and D-D ($\overset{-}{x}$±s, n=10).

Group	Dose	FIB(g/L)	D-D(ng/mL)
Normal control	----	2.70±0.07	332.09±8.43
Model control	----	2.83±0.11^#^	341.38±8.05^#^
Positive control	30 mg/kg	2.75±0.05	322.88±8.56^*∗∗∗*^
Low dose	7 mg/kg	2.68±0.12^*∗∗*^	331.87±8.76^*∗*^
Middle dose	14 mg/kg	2.56±0.11^*∗∗∗*^	328.12±6.50^*∗∗*^
High dose	35 mg/kg	2.76±0.13	347.05±6.58

Note: Data are presented as mean±S.E.M. for each group. ^#^*P<0.05*: significant difference compared with normal control group. ^*∗*^*P<0.05*, ^*∗∗*^*P<0.01, *and^*∗∗∗*^*P<0.001*:significant difference compared with model control group.

## Data Availability

The data used to support the findings of this study are included within the article.
